# Peroxisome Proliferator-Activated Receptors in the Modulation of the Immune/Inflammatory Response in Atherosclerosis

**DOI:** 10.1155/2008/285842

**Published:** 2008-08-21

**Authors:** Ana Z. Fernandez

**Affiliations:** Laboratorio de Hemostasia y Genética Vascular, Centro de Biofísica y Bioquímica, Instituto Venezolano de Investigaciones Científicas (I.V.I.C.), 1020 Caracas, Venezuela

## Abstract

Inflammation has been recognized as an important hallmark of atherosclerosis. The pharmacological activation of PPAR-*γ* by the thiazolidinediones in diabetes, and of PPAR-*α* by the fibrates in hyperlipidemia has been shown to help to reduce inflammatory markers in preclinical and clinical studies. PPARs are known to modulate immune pathways through at least three different mechanisms: by direct binding to PPRE of anti-inflammatory cytokines genes; by transrepression of transcription factors like NF-*κ*B and AP-1; or by corepression. The regulation of the inflammatory pathways by PPARs can be achieved on each one of the cells involved in the atherosclerotic process, that is, monocytes, macrophages, T cells, endothelial cells, and smooth muscle cells. Moreover, as each of these cellular components is interconnected with each other, PPAR activation in one cell type could affect the other ones. As activation of PPARs has clear ant-inflammatory benefits, PPARs ligands should be considered as a new therapeutical approach to ameliorate the exacerbated immune response in atherosclerotic diseases.

## 1. INTRODUCTION

Cardiovascular diseases represent the main cause of morbidity and mortality in western societies since the 20th century and mostly are a consequence of atherosclerosis, a prior established pathology. Atherosclerosis is defined as a progressive, chronic pathology characterized by the accumulation of lipids and fibrous elements in the large arteries. It is classified as an inflammatory disease, since in every phase of the atherosclerotic process, the immune response has a significant role [1]. Immune cells like monocytes, macrophages, and T-cells are crucial in the development of the atheroma and the stimulation and activation of endothelial cells (EC) and smooth muscle cells (SMC), cellular components of the vascular wall are extremely relevant for the recruitment of the cells responsible for the immune response.

Peroxisome proliferator-activated receptors (PPAR) and their agonists have been gaining more attention recently in regard to the study of the mechanisms involved in the etiology and pathogenesis of atherosclerosis. The expression of PPAR-*α*, -*δ*/*β*, and -*γ* in vascular wall cells and in immune cells, as well as in atherosclerotic lesions, has been described [2, 3]. The pharmacological modulations of both PPAR-*α* and -*γ* as therapeutic treatments for diabetes and hyperlipidemia have been linked to an improvement on the low-grade inflammation associated with these conditions [4, 5]. The inhibition of function and/or expression of certain genes critical for the initiation or maintenance of inflammatory cells recruitment, survival, proliferation, and activation has been shown to alter the progression of atherosclerotic lesions [6]. In this regard, the three PPAR isoforms certainly could play distinctive roles in modulating the inflammatory response in atherosclerosis.

## 2. PPAR: GENERAL CHARACTERISTICS

The PPARs belong to a subfamily of the nuclear receptors superfamily and are ligand-activated transcription factors which heterodimerize with the retinoic X receptor and recognize PPAR response elements (PPRE) localized in the promoter region of target genes [2]. In addition to the direct involvement of PPAR in the gene-specific transcription, PPAR could also repress the transcription of certain genes brought about by the proinflammatory transcription factors, nuclear factor (NF)-*κ*B, activation protein-1 (AP-1), and signal transducer and activator of transcription 1 (STAT-1), through the binding and sequestration of their corresponding cofactors [7].

Three PPAR forms have been described: PPAR-*α* (NR1C1), PPAR-*δ*/*β* (NR1C2), and PPAR-*γ* (NR1C3). These different receptors show a similar protein structure in spite of their different coding genes [3]. PPAR-*α* and PPAR-*γ* have been recognized to be key players in both cellular differentiation processes and anti-inflammatory regulation and, most recently, PPAR-*δ*/*β* has also been implicated in the immune response [8].

PPAR-*α* is mainly expressed in tissues characterized by a high rate of fatty acid catabolism (liver, kidney, heart, and muscle) and is the most abundantly expressed PPAR isoform in human endothelial cells (EC) [9]. The transcriptional activity of PPAR-*α* is stimulated by a variety of compounds (see Table 1) [2]. PPAR*α* synthetic ligands, such as clofibrate, fenofibrate, and bezafibrate, were developed as hypolipidemic agents, through optimization of their lipid-lowering activity in rodents, even before the discovery of the PPARs [10]. PPAR-*α* is involved in the control of lipoproteins metabolism, fatty acid oxidation, and in the cellular uptake of fatty acids [10]. Studies in vitro suggest that PPAR-**α** also regulates the expression of genes that control inflammatory responses in EC, SMC, and macrophages, cells known to be implicated in the inflammatory response of vascular EC and in the pathology of atherosclerosis [9, 11].

PPAR-*δ*/*β* is ubiquitously expressed both in vascular SMC as well as in EC, besides liver, kidney, and abdominal adipose tissues. Several eicosanoids have been reported to activate PPAR-*δ*/*β*, including PGA_1_ and PGD_2_, and a synthetic prostaglandin carbaprostacyclin [10]. The important physiological roles of PPAR-*δ*/*β* are highlighted in genetically modified mouse models as deletion of PPAR-*δ*/*β* in mice leads to incomplete but very high penetration of a lethal phenotype and PPAR-*δ*/*β* heterozygous animals display abnormal wound healing [12]. Additionally, PPAR-*δ*/*β* has been implicated in the maintenance of lipid homeostasis [13], keratinocyte proliferation in response to injury [12, 14, 15], hyperplasic development of adipose tissue in animal under a high-fat diet [16], and was recently shown to have beneficial effects on muscle fat oxidation and lipid profiles in humans [17, 18].

The best characterized receptor in this group of nuclear factors is the PPAR-*γ* which plays a significant role in adipocyte differentiation and fat deposition [10, 19]. This receptor is expressed in adipose tissue, skeletal, and cardiac muscle and is also expressed in human peripheral blood monocytes and in monocytic cell lines. The large list of activators of PPAR-*γ* includes prostaglandin-derived 15-deoxi-delta 12,14PGJ_2_ (15d-PGJ_2_), the thiazolidinediones (TZD) troglitazone (Rezulin^R^), pioglitazone (Actos^R^), and rosiglitazone (Avandia^R^), among others (see Table 1) [2, 10, 20]. The currently marketed TZDs are potent and selective PPAR-*γ* activators; they are antidiabetic agents that increase the insulin sensitivity of target tissues in animal models of non-insulin-dependent diabetes mellitus and in diabetic patients.

One of the features that characterize the PPAR is the large amounts of natural and synthetic molecules that can activate them. PPARs are differentially activated by naturally occurring eicosanoids and related molecules [21, 22]. Nitroalkene derivatives of fatty acids have also been characterized as endogenous PPAR ligands. Schopfer et al. [23], using CV-1 reporter cells cotransfected with plasmids containing the ligand-binding domain for PPAR-*α*, -*δ*/*β*, and -*γ*, found that nitrated linoleic acid (1 *μ*M) (LNO_2_) was capable to induce significant activation of PPAR-*γ* (620%), PPAR-*α* (325%), and PPAR-*δ*/*β* (221%), when compared to control cells. Concomitant works revealed the existence of LNO_2_ and other fatty acid nitration products, generated by NO-dependent reactions, in human red cells, blood, and urine samples [24, 25].

The general approach used to study the effects of PPARs is through PPAR activation by natural or synthetic agonist. However, PPAR ligands have been shown to have both PPAR-dependent and -independent actions, which could be addressed by in vivo genetic manipulation, such as PPAR-knockout animals or in cell-based systems using small interfering RNA [26].

## 3. INFLAMMATION IS MODULATED BY Ox-LDL THROUGH PPAR

A primary initiating event in atherosclerosis is the accumulation of modified low-density lipoprotein (LDL) in the subendotelial matrix, such as oxidized-LDL (ox-LDL. These ox-LDL are taken up by macrophages, inducing the formation of foam cells, and stimulating the EC to produce a number of proinflammatory molecules, such as monocyte chemotactic protein (MCP)-1, whose effects are mediated by the G protein-coupled receptor CCR2, expressed mainly in monocytes, basophils, and certain subsets of T cells [27, 28].

One of the most studied factors involved in the atherosclerotic process is ox-LDL [29]. Ox-LDL provides ligands for PPAR-*γ* and PPAR-**α** [30, 31] and also seems to enhance the expression of PPAR-*γ* in differentiated macrophages [32]. Ox-LDL, oxidized linoleic acid, and metabolites derived from it, including 9-hydroxyoctadecaenoic acid (HODE) and 13-HODE, induce PPAR-*γ* activation in monocytes and monocytic cellular lines, stimulating the transcription of the ox-LDL receptor CD36/fatty acid translocase, through a PPRE in the promoter of CD36/fatty acid translocase gene, which leads to the formation of foam cells [19, 30, 33].

The first contact between ox-LDL and monocyte/macrophage cell elicits reactive oxygen species (ROS) formation, followed by a desensitization of macrophages via activation of PPAR-*γ*, which reduces ROS production, giving the ox-LDL a dual role in the activation/deactivation of macrophages [34]. Ox-LDL inhibited NF-*κ*B-mediated IL-12 production in LPS-stimulated mouse macrophages, involving both inhibition of the NF-*κ*B-DNA interactions and physical interactions between NF-*κ*B and PPAR-*γ* [35]. Activation of NF-*κ*B is involved in the pathophysiology of many inflammatory chronic diseases, including atherosclerosis. Binding sites for NF-*κ*B have been found in cellular adhesion molecules and chemokines [36, 37]. The NF-*κ*B signaling pathway is activated by the proinflammatory cytokines TNF-*α* and IL-1*α* which are the major cytokine inducers of gene expression in EC. In resting macrophages, PPAR-*γ* ligands completely blocked the ox-LDL-mediated activation of NF-*κ*B [38].

PPAR-*α* activation by ox-LDL in the vascular wall components seems to upregulate the inflammation. The activation of this receptor in human EC by oxidized components in LDL resulted in an increase in the production of chemotactic factors for monocytes (MCP-1 and IL-8), conferring it a proinflammatory effect [9].

## 4. MOLECULAR MECHANISMS FOR THE REGULATION OF INFLAMMATORY/IMMUNE RESPONSE IN ATHEROCLEROSIS BY PPAR AGONISTS

The adhesion of monocytes to the vascular wall is mediated by adhesion molecules expressed on the surface of the EC, such as vascular cell adhesion molecule-1 (VCAM-1), intercellular adhesion molecule 1 (ICAM-1), P and E selectins [39], which is a phenotypic hallmark of EC activation and a critical step of many proinflammatory processes. The constitutive activation of PPAR-*γ* in ECs inhibited the expression of VCAM-1, ICAM-1, and E-selectin, by interference with NF-*κ*B and AP-1 transactivation [40].

Many of the immune cells in the atheroma exhibit signs of activation and produce proinflammatory cytokines. In addition to monocytes and macrophages, T cells definitely play a significant role in the lesion, where CD4+ T cells dominate over CD8+ cells. CD4+ T cells differentiate into several subtypes, being T helper-1 cells (Th1) the predominant pattern in atherosclerosis. IFN-*γ* is a major proatherogenic Th1 cytokine, promoting macrophage and endothelial activation with production of adhesion molecules, cytokines, chemokines, radicals, proteases, and coagulation factors. In addition, IFN-*γ* inhibits cell proliferation, collagen production, and cholesterol efflux [41]. PPAR-*α* and PPAR-*γ* mRNA, and protein are expressed in isolated human CD4+ T cells, and the activation of each one of them by specific ligands reduces the secretion of IFN-*γ*, TNF-*α*, and IL-2 in these lymphocytes [42]. Furthermore, the effect of PPAR agonist on CD4+ T cells impaired their action on monocytes and EC, suggesting that PPAR modulation of inflammatory pathways in T cells may offer clinical benefits in atherosclerosis [43].

TNF*α* is a catabolic proinflammatory cytokine, produced by Th1 cells and macrophages, that exerts a wide range of effects on cells and tissues, through the activation of the transcription factor NF-*κ*B. Gene targeting of TNF*α* leads to reduced atherosclerosis [42]. It has been shown that human aortic EC activation by TNF*α* could be prevented by incubation with MCC-555, a novel TZD, while pioglitazone and rosiglitazone did not [44].

PPAR-**α** has been shown to inhibit transcriptional responses to inflammatory stimuli by interfering with the activation of NF-**κ**B, leading to the reduction of VCAM-1 in EC [11]. In vascular SMC, PPAR-**α** agonists inhibited IL-1-induced IL-6 expression, cyclooxygenase-2 (COX-2) and prostaglandin production [45]. The upregulation of antioxidant enzymes activity by the PPAR-*α* activators reduced the oxidative stress and, as the result, it might inhibit the NF-*κ*B activation and subsequent inflammatory response [46].

The regulation of chemokine-receptor expression may be a crucial mechanism to control monocyte responses to chemokines. Monocytic-line THP-1 cells incubated with rosiglitazone reduced CCR2 surface expression by about 50–60% (*P* < .001) compared with untreated control cells [29]. PPAR-*γ* agonists suppress monocyte elaboration of inflammatory cytokines TNF*α*, IL1, and IL6 [47]. Table 2 summarizes the general effects of PPAR activation on each cell type.

In murine hypercholesterolemic models, the administration of PPAR-*γ* ligands inhibited the development of atherosclerosis, in spite of the high expression of CD36 in the vascular wall [48]. Downstream PPAR*γ*-dependent anti-inflammatory effects of 15d-PGJ_2_ include the inhibition of transcriptional activation by NF-*κ*B via I*κ*B, which affect gene expression of inducible nitric oxide synthase (iNOS), TNF*α*, COX-2, IFN-*α*, IL-1, IL-6, and LPS-induced transcription of AP-1 and STAT-1 [49]. Oxidative burst in macrophages is also attenuated by PPAR-*γ* activation [50].

Other regulatory mechanism that could be attributed to PPAR is the selective activation of anti-inflammatory cytokines, like IL-10. IL-10 has potent deactivating properties in macrophages and T cells and modulates many cellular processes that may interfere with the development and stability of the atherosclerotic plaque [51]. Using nanomolar concentrations of rosiglitazone, Thompson et al. have demonstrated the upregulation of IL-10, likely through a functional PPRE found in the promoter region of IL-10 gene [52].

Major histocompatibility complex class II molecules (MHC-II) play a critical role in the induction of immune responses by presenting peptides of foreign antigens to CD4+ T lymphocytes, which result in their activation and proliferation. Human ECs are capable of expressing MHC-II under treatment with IFN-*γ* and this induction is repressed by PPAR-*γ* ligands [53].

PPAR-*δ*/*β* seems to have dual effects in regard to inflammation in atherosclerotic models [8, 54–57]. Although results from both PPAR-*δ*/*β*−/− and PPAR-*δ*/*β* overexpressing macrophages suggested a proinflammatory role for PPAR-*δ*/*β*, treatment of cells with PPAR-*δ*/*β* agonist GW501516 suppressed the expression of MCP-1 and IL-1*β* in a receptor-dependent manner, indicating that activation of PPAR-*δ*/*β* had an anti-inflammatory effect [54]. The pharmacological modulation of PPAR-*δ*/*β* in atherosclerotic LDLR−/− mice showed decreased expression of MCP-1, TNF*α*, and ICAM-1 [55] and similarly, proinflammatory modulators were suppressed in apoE−/−mice treated with GW501516 [56]. Fan et al. also found an anti-inflammatory effect of PPAR-*δ*/*β* agonists in TNF*α*-activated EC [8]. In addition, Takata et al. have found that PPAR-*δ*/*β* agonist GW0742 substantially inhibited vascular proinflammatory gene expression, macrophage content, and atherosclerosis in an angiotensin II-induced high fat-fed male LDLR−/− mouse model of accelerated atherosclerosis [57]. Furthermore, promising results were obtained in a clinical evaluation of the PPAR-*δ*/*β* agonist GW501516 in six obese males [17]. Although inflammatory markers were not considered in this study, the wide range of beneficial effects by the pharmacological activation of PPAR-*δ*/*β* could suggest an improvement on the inflammatory grade of proatherogenic conditions and an attractive therapeutic target for drug development to treat atherosclerosis [56].

Post-translational modifications have been found to modulate transcriptional activity of PPAR-*γ* [57]. One of these modifications is sumoylation, the covalent attachment of a small ubiquitin-like proteins (Ubl) called SUMO-1. Pascual et al. [58] proposed a novel pathway mediating ligand-dependent transrepression of inflammatory response genes by PPAR-*γ* in macrophages which involves ligand-dependent sumoylation of the PPAR-*γ* ligand-binding domain. This targets PPAR-*γ* to nuclear receptor corepressor (NCoR)/histone deacetylase-3 (HDAC3) complexes on inflammatory gene promoters, which in turn prevents recruitment of the ubiquitylation/19S proteosome machinery that normally mediates the signal-dependent removal of corepressor complexes required for gene activation. As a result, NCoR complexes are not cleared from the promoter and target genes are maintained in a repressed state. This mechanism provides an explanation for how an agonist-bound nuclear receptor can be converted from an activator of transcription to a promoter-specific repressor of NF-*κ*B target genes that regulate immunity and homeostasis [59].

## 5. CONCLUSION

Basic and clinical research points out towards an intrinsic interplay between immune/inflammatory mediators and PPAR activation in the pathogenesis and development of atherosclerosis. Each one of PPARs seems to have and share different output in order to reach the cellular homeostasis. It could be seen that Inflammation, as a disruption of homeostasis, has not only internal control by its own but an external regulation through the activation of PPARs. However, it is still not clear what are the “real functional in vivo” natural ligands of PPAR. Meanwhile, the search for the ideal synthetic ligand that would combine the beneficial effects of PPAR activation is ongoing. Such “magical” drug would be prescribed either as hypoglycemic, hypolipemic and anti-inflammatory agent, but would need to be chronically administered. Overall, pharmacological activation of PPARs might be a better approach to cover all the underlying inflammatory features of the atherosclerosis.

## Figures and Tables

**Table 1:** Properties and agonists of PPARs.

Characteristic	PPAR-*α*	PPAR-*γ*	PPAR-*δ*/*β*
General distribution	Liver, heart, kidney, muscle. Endothelial cells	Heart, intestine, kidney, pancreas, spleen, muscle, adipose tissue	Liver, intestine, kidney, abdominal adipose tissue, skeletal muscle
Natural ligands	Saturated and unsaturated fatty acids; arachidonic acid-derived eicosanoids from the lipoxygenase pathway: 8-S-hydroxyeicosatetraenoic acid and leukotriene B4; insulin; oxidized LDL	Linoleic acid, linolenic acid, arachidonic acid, eicosapentenoic acid, 15-deoxy Δ12, 14-prostaglandin J2; 15-LOX metabolites (9-HODE and 13-HODE)	Saturated and unsaturated fatty acids; eicosanoids: PGA_1_ and PGD_2_
Synthetic agonists	Hypolipidemic fibrate drugs: fenofibrate, genfibrozil; plasticizers, ureidofibrates; WY14643, JTT-501, GW-2331 and PD72953	Antidiabetic thiazolidinediones: pioglitazone, troglitazone, rosiglitazone (BRL-49653), MCC-555; isoxazolidinedione JTT-501; tyrosine-based agonist: GI2-62570, GW-1929, and GW-7845 from GSK; *α*-alcoxy-*β*-phenylpropanoic acid; weak agonist: LTD4 receptor antagonist LY-171883; COX inhibitors: indomethacin, ibuprofen, fenoprofen, and flufenamic acid. Docosohexanoic derivatives	Leukotriene antagonist L-165041; phenylacetic derivatives L-796449 and L-783483; GW-2433, GW-501516, GW0742X; carbaprostacyclin
Gene/protein expression affected	Lipoprotein lipase, Apo CIII, Apo AI, Apo AII, fatty acid transporter protein, Acyl-CoA synthetase, mitochondrial HMG-CoA synthase, mitochondrial uncoupling protein 1	Acyl-CoA synthetase, fatty acid transporter protein, CD 36, lipoprotein lipase, TNF-*α* mitochondrial uncoupling protein 1–3; insulin-dependent glucose transporter 4	Fatty acid transporter protein, CD 36, fatty acid translocase, adipocyte lipid-binding protein, ABCA1, 14-3-3*ε*

**Table 2:** PPAR-mediated immune downregulation in monocytes/macrophages, T cells, EC, and SMC.

Monocyte/macrophage	T cell	EC	SMC
Reduced expression of TNF-*α*, CCR-2, synthesis of IL-6, IL-1 by PPAR-*γ*. Oxidative burst suppressed by PPAR-*γ*.	Reduced expression of TNF-*α*, IFN-*γ*, IL-2 by activation of PPAR-*γ* and -*α*.	Reduced expression of VCAM-1, ICAM-1, E-selectin and MHC-II by PPAR-*γ*, -*α*, and -*δ*/*β*; impaired TNF-*α* activation.	Reduced synthesis of IL6, COX-2, and prostaglandin by PPAR-*α*.

## ABBREVIATIONS

ABC: ATP-binding cassette
ALBP: Adipocyte lipid binding protein
AP-1: Activation protein-1
COX: Cyclooxygenase
EC: Endothelial cells
FAT: Fatty acid translocase
HDAC3: Histone deacetylase-3
HDL: High-density lipoprotein
HMG-CoA: Hydroxy methyl glutaryl coenzyme A
HODE: Hydroxy octadecaenoic acid
ICAM-1: Intercellular adhesion molecule 1
IFN: Interferon
IL: Interleukin
LDL: Low-density lipoprotein
LNO_2_: Nitrated linoleic acid
LPS: Lipopolysaccharide
MCP: Monocyte chemotactic protein
MHC-II: Major histocompatibility complex class II molecules (MHC-II)
NCoR: Nuclear receptor corepressor
NF-*κ*B: Nuclear factor *κ*B
NOS: Nitric oxide synthase
PG: Prostaglandin
PPAR: Peroxisome proliferator-activated receptors
PPRE: PPAR response elements
ROS: Reactive oxygen species
SMC: Smooth muscle cells
STAT-1: Signal transducer and activator of transcription 1
Th: T helper cells
TNF: Tumor necrosis factor
TZD: Thiazolidinediones
Ubl: Ubiquitin-like proteins
VCAM-1: Vascular cell adhesion molecule-1.
